# Eating, Drinking and Exercise

**Published:** 1881-12

**Authors:** 


### EATING, DRINKING AND EXERCISE.

WE find that the law of supply and demand is just as operative in regard to each individual as to communities or nations. Allowing some "lee-way" for the adaptability of the system to abuses and accidents, nature demands that the quality and quantity of food taken shall exactly meet the demands imposed by labor or exercise. If necessary supplies are not furnished to meet these demands, drafts are made on the energy that is stored in the system, and debility results. If the supply of nutriment to the stomach is in excess of the demand, such excess is a source of danger. Men seldom govern themselves by the same rules that they apply to their domestic animals. A horse is fed according to the amount of labor required of him. He has no option as to the quantity, though in many instances it would be far better if he had. There are men who think that any food is good enough for them, and the less they can manage to get along with the better. There are others who think of little but eating and drinking. Either extreme is to be avoided. Inadequate nourishment results in debility and premature death. Habitual excesses in eating and drinking often bring on disease by increasing the quantity of the blood beyond the power of the heart to keep it in circulation, and thus it is that such persons are apt to die suddenly of incurable disease of some vital organ. The attainment of old age under either of the above conditions is simply impossible. He who would live to a purpose, and enjoy life with the prospect of a serene and tranquil old age; who would, when nearing the close of life, regard the inevitable change without fear, and without remorse, must in all things avoid extremes.

